# Planar Cell Polarity Enables Posterior Localization of Nodal Cilia and Left-Right Axis Determination during Mouse and *Xenopus* Embryogenesis

**DOI:** 10.1371/journal.pone.0008999

**Published:** 2010-02-02

**Authors:** Dragana Antic, Jennifer L. Stubbs, Kaye Suyama, Chris Kintner, Matthew P. Scott, Jeffrey D. Axelrod

**Affiliations:** 1 Department of Pathology, Stanford University School of Medicine, Stanford, California, United States of America; 2 Departments of Developmental Biology, Genetics, and Bioengineering, Howard Hughes Medical Institute, Stanford University School of Medicine, Stanford, California, United States of America; 3 The Salk Institute for Biological Studies, La Jolla, California, United States of America; Texas A&M University, United States of America

## Abstract

Left-right asymmetry in vertebrates is initiated in an early embryonic structure called the ventral node in human and mouse, and the gastrocoel roof plate (GRP) in the frog. Within these structures, each epithelial cell bears a single motile cilium, and the concerted beating of these cilia produces a leftward fluid flow that is required to initiate left-right asymmetric gene expression. The leftward fluid flow is thought to result from the posterior tilt of the cilia, which protrude from near the posterior portion of each cell's apical surface. The cells, therefore, display a morphological planar polarization. Planar cell polarity (PCP) is manifested as the coordinated, polarized orientation of cells within epithelial sheets, or as directional cell migration and intercalation during convergent extension. A set of evolutionarily conserved proteins regulates PCP. Here, we provide evidence that vertebrate PCP proteins regulate planar polarity in the mouse ventral node and in the *Xenopus* gastrocoel roof plate. Asymmetric anterior localization of VANGL1 and PRICKLE2 (PK2) in mouse ventral node cells indicates that these cells are planar polarized by a conserved molecular mechanism. A weakly penetrant *Vangl1* mutant phenotype suggests that compromised *Vangl1* function may be associated with left-right laterality defects. Stronger functional evidence comes from the *Xenopus* GRP, where we show that perturbation of VANGL2 protein function disrupts the posterior localization of motile cilia that is required for leftward fluid flow, and causes aberrant expression of the left side-specific gene *Nodal*. The observation of anterior-posterior PCP in the mouse and in *Xenopus* embryonic organizers reflects a strong evolutionary conservation of this mechanism that is important for body plan determination.

## Introduction

In the mouse, left-right asymmetry has been proposed to be controlled by fluid flow in the node propelled by clockwise movement of motile nodal cilia [1]–[5]. Leftward nodal flow depends on positioning of motile cilia at the posterior sides of nodal cells, causing an asymmetric stroke that produces the directional movement of the nodal fluid [2], [3]. This flow begins at the 1 to 2 somite stage [2]. Both mechanical effects of flow, and flow-dependent leftward transport of signaling molecules, have been proposed to set in motion asymmetric gene expression patterns that establish the laterality of subsequently developing structures [2]–[4].

The asymmetric signal in the mouse ventral node initiates Nodal and Lefty signaling on the left side of the node and in the left lateral plate mesoderm (LPM) [2], [3]. Nodal then induces expression of the homeobox gene *Pitx2* that regulates asymmetric heart, gut, and lung morphogenesis [6], [7]. While left-sided expression of *Lefty* and *Nodal* is transient, asymmetric expression of *Pitx2* in LPM is maintained until E8.5 (8–9 somite stage) [6]. Later in development, at stages E9.5 and E10.5, asymmetric distribution of *Pitx2* is observed on the left side of the heart, foregut primordia, and lung buds [6], [7]. In mutants with L-R asymmetry defects, such as *iv/iv* embryos, the expression of *Pitx2* is either reversed, bilateral, or absent [6], [7].

In *Xenopus*, the gastrocoel roof plate (GRP) is homologous to the mouse ventral node and has a monociliated planar polarized epithelium. As in the mouse node, the cilia are motile, they are posteriorly located, and they rotate in a clockwise fashion thus generating leftward fluid flow. In both species, Inversin, Polycistin-2, and Dynein heavy chain proteins are important for ciliary structure, motility, and laterality determination [1], [8]–[10]. However, in neither system, is it known how the posterior localization and tilt of the motile cilia is established.

Planar polarization of cells is required in a variety of tissues during vertebrate development, and its disruption may lead to developmental anomalies including open neural tube defects, polycystic kidneys, conotruncal heart defects and deafness [11]–[14]. A number of genes, most first identified in Drosophila, are known to regulate PCP dependent processes. PCP signaling has been extensively studied in *Drosophila* using genetic and protein-localization approaches, revealing a tight link between asymmetric cortical distribution of PCP proteins and their PCP signaling functions [14]–[16]. Similar studies in mice showed that the mammalian PCP proteins Dishevelled (DVL2, DVL3), Frizzled (FZ3, FZ6), Van Gogh like (VANGL2; originally called Looptail), and PRICKLE2 (PK2) [11], [12] [17]–[22] also localize asymmetrically in tissues that display planar polarity, such as the inner ear sensory epithelium. As in flies, PK2 and FZ6 accumulate on opposite sides of cells [21]. These findings suggest that the PCP signaling mechanism in mammals is similar to that in flies [14], [15], [23]. In both insects and vertebrates, PCP proteins influence cytoskeletal rearrangements that lead to specific orientation of morphologically polarized cells within epithelial sheets[24]. Thus the PCP mechanism is an attractive candidate for determining the posterior localization and tilt of motile cilia in the mouse ventral node and in the *Xenopus* GRP.

## Results

**Figure 1:**
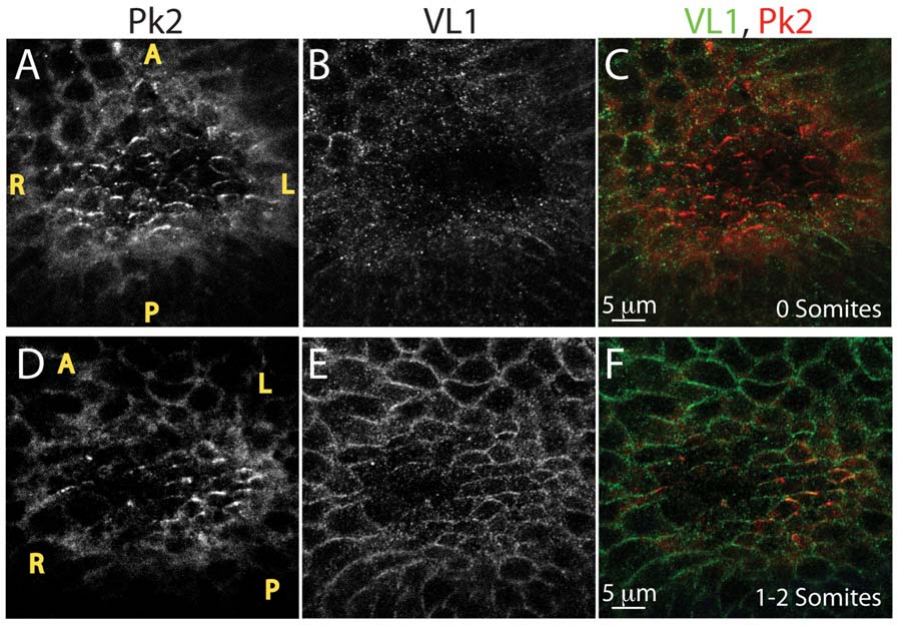
VANGL1 and PRICKLE2 set up PCP in the mouse ventral node. (**A–F**) Localization of VL1 and PK2 proteins in the ventral node: PRICKLE2 is expressed in the node of 0 somite embryos (**A**, **C** red) prior to VANGL1, which is detected in node cells (**E, F**, green) of 1–2 somite embryos. VL1 and PK2 co-localize in node cells and form crescents pointed toward the anterior (**F**, yellow). Motile cilia can be visualized above the plane of VL1 or PK2 localization (not shown). Yellow letters mark anterior (A), posterior (P), Left (L) and right (R). As nodes were imaged from the ventral side, left side of the embryo is on the right side of each panel and right side of the embryo is on the left side of each panel.

We hypothesized that PCP may be required to position the nodal cilia in the posterior of each cell. If so, nodal cells must be polarized prior to the onset of nodal flow. To determine whether a conserved PCP signaling mechanism may establish PCP in the mouse ventral node, and if so, when this polarity is established, we used VANGL1, PK1, and PK2 antibodies to examine expression and localization of these proteins in early headfold stage embryos with 0 somites, prior to initiation of nodal flow, and in embryos with 1 to 2 somites, the time the nodal flow begins (**Fig. 1**). PK2 asymmetric distribution at anterior-posterior boundaries of central cells in the ventral node can be detected in very early headfold 0 somite embryos (**Fig. 1A, C**), presumably prior to the beginning of nodal flow. Asymmetric distribution of VANGL1 was also detected, but the earliest we detected it was later, at the 1 to 2 somite stage (**Fig. 1B, C, E, F**), the time when nodal flow begins. At this time, PK2 and VANGL1 colocalize. Both proteins appear to be at the anterior side of the cells, although mosaic studies will be needed to confirm this. Localization of these two PCP proteins in crescents is reminiscent of that seen in *Drosophila* tissues, and of PCP protein localization patterns in the vertebrate sensory epithelia of the inner ear [11], [17], [18], [20], [21]. These observations reveal that the cells of the node exhibit molecular PCP along the anterior-posterior axis, and strongly suggest that the conserved PCP signaling system is engaged to polarize these cells along the anterior-posterior axis. The relevance of PK2 asymmetric distribution in crescents preceding that of VANGL1 is not clear, but might suggest that PK2 could be involved in establishing PCP while VANGL1 may be necessary to maintain it. We did not detect expression of PK1 in the ventral node (not shown), in agreement with reported *in situ* hybridization results [25].

To investigate the potential functional requirement for PCP signaling in vertebrate left-right asymmetry, we generated a *Vangl1* mutant mouse using BayGenomics *Vangl1* gene-trap ES cells, as has been done by another group [26] (**Fig. S1**). We similarly refer to the allele as *Vangl1gt.* In our animals of mixed genetic background, many *Vangl1gt/gt* homozygotes were viable and fertile as previously reported [26]. Also as previously reported, we found that the two *Vangl* genes, *Vangl1* and *Vangl2*, genetically interact to control PCP. *Vangl1+/gt, Vangl2+/Lp* doubly heterozygous mice had severe open neural tube defects as well as moderate PCP defects in cochlea (**Fig. S1, S2**), not present in either of the *Vangl* heterozygotes. In addition, we observed that at low penetrance, some homozygous *Vangl1gt/gt* mutants died as early embryos. The variable viability of our *Vangl1gt/gt* homozygous animals prompted us to carefully examine adult homozygotes for the expression of *Vangl1-βGeo* (the predicted mutant transcript that has β-Galactosidase-Neomycin fusion [*βGeo*] spliced after exon 3), *Vangl1* WT mRNAs, and their corresponding proteins. Viable animals, homozygous for the gene-trap insertion, produced low but variable amounts of both *Vangl1-βGeo* and *Vangl1* WT mRNAs and proteins (**Fig. S3**), suggesting that alternative splicing creates small amounts of functional *Vangl1* transcripts and protein that are sufficient to suppress the homozygous null phenotype in the majority of individuals.

**Figure 2:**
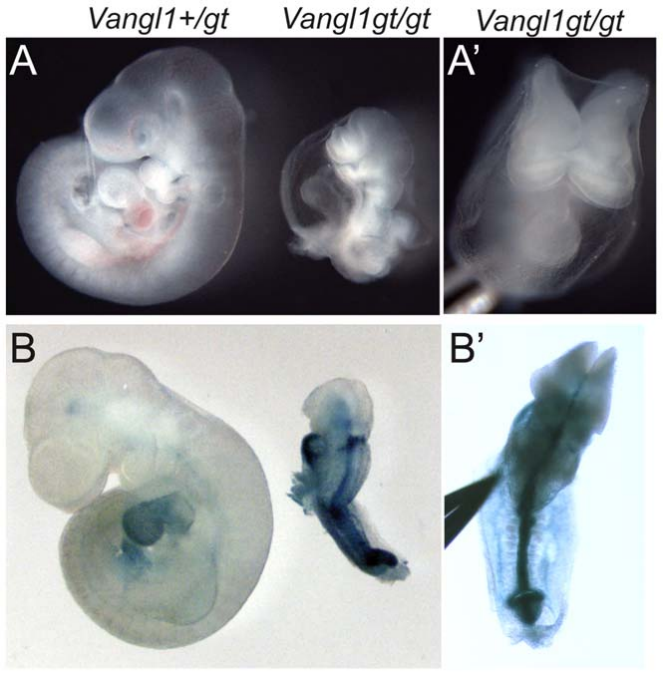
*Vangl1gt/gt* homozygous embryos fail to turn. *Vangl1+/gt* heterozygous embryos are normal and they develop into viable, fertile adults. In contrast, ∼14% of *Vangl1gt/gt* mutants die at E9.5–10.5. (**A'** **, B'**) Cranial (A') and dorsal (B') views of *Vangl1gt/gt* mutant embryos shown in **A** and **B** showing open neural tube defects. (**B, B'**) One mutant and its heterozygous littermate were stained for VANGL1-βGEO fusion protein. Each of five phenotypically mutant *Vangl1gt/gt* homozygotes isolated at this stage failed to turn from lordotic to fetal position.

**Figure 3:**
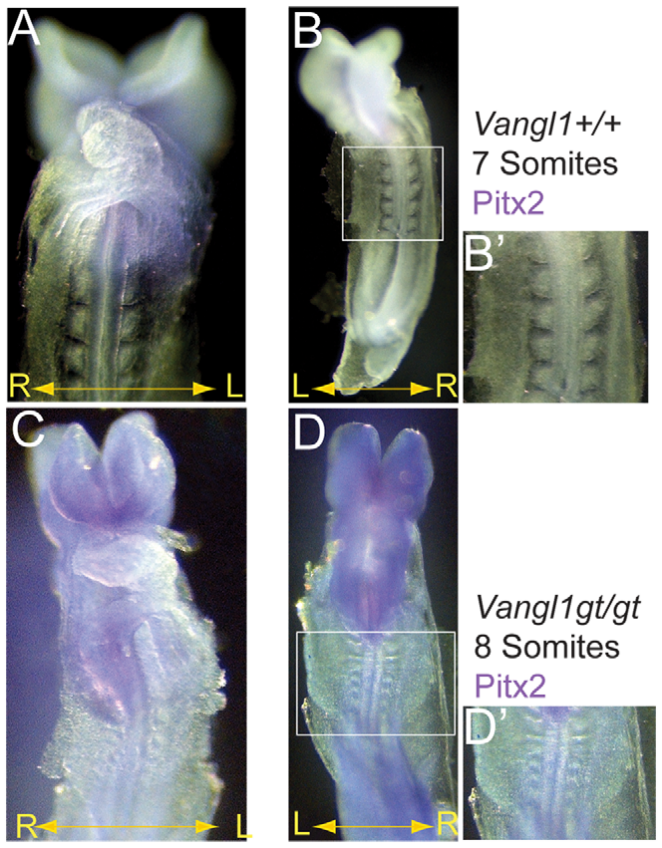
Aberrant *Pitx2* expression in a *Vangl1gt/gt* homozygous embryo. (**A, B**) Wild-type embryos with 7 somites (**A, B, B'**) have Pitx2 expressed in the left lateral plate mesoderm (LPM). (**C, D**) A *Vangl1gt/gt* homozygous embryo with 8 somites has Pitx2 expressed bilaterally but predominant expression is in the right LPM. (**D'**) Somites of the *Vangl1gt/gt* homozygous mutant are narrow and compressed in the anterior-posterior direction, in comparison to the wild-type embryo (**B'**). The mis-expression of *Pitx2* and the turning defect suggest that *Vangl1* might regulate L-R asymmetry establishment in the mouse ventral node. (**B'**) and (**D'**) are the boxed regions from (**B**) and (**D**) and are at the same magnification.

Analysis of animals from timed pregnancies revealed that 14% of *Vangl1gt/gt* homozygotes died early in development, between E9.5 and E10.5, and they displayed varying degrees of known PCP-related defects (**Fig. 2**). Each of five phenotypically mutant *Vangl1gt/gt* embryos we obtained at this stage had an open neural tube (**Fig. 2**), and the somites were narrow in the anterior-posterior direction and tended to be elongated medio-laterally compared to wild type, reminiscent of PCP dependent convergent extension defects (****Fig, 3B**', D'** and not shown) [11], [27]. Embryos with these phenotypes were never recovered among heterozygous siblings, or from crosses between wild-type, BL6 or 129Ola animals. In addition, despite advancing to the 20–25 somite stage and maintaining viability up to E9.5-E10 as judged by beating hearts at the time of isolation, each of the phenotypically mutant embryos failed to turn. The turning of mouse embryos is a process that converts the embryo from lordotic to fetal position at the 8- to 13-somite stage of development [28]. Turning defects are often associated with aberrant L–R patterning in the mouse ventral node [1], [2], [5]. Consistent with this interpretation, *Pitx2* in two phenotypically mutant *Vangl1gt/gt* embryos isolated at E8.5 (**Fig. 3**) and E10 (not shown) was bilateral, with predominant expression on the right side of the embryo, consistent with aberrant L–R signaling. Normal left-sided expression of *Pitx2* was observed in seven additional *Vangl1gt/gt* embryos with normal morphology that were obtained from these litters, as well as in all the embryos from five other litters from *Vangl1gt/+* parents, from which we expect Mendelian ratios of *Vangl1gt/gt* homozygotes, although they were not genotyped. Therefore, abnormal *Pitx2* expression appears to correlate closely with aberrant turning. While the abnormal turning and *Pitx2* phenotypes, observed in a fraction of *Vangl1gt/gt* homozygous mutant mice, hint at a left-right lateralization function for *Vangl1*, the rarity of isolating phenotypically mutant embryos prevents us from doing a thorough analysis, and thus must be considered only suggestive of such a function. However, striking polarized distribution of VANGL1 and PK2 proteins in central node cells suggests that PCP signaling establishes anterior-posterior polarity and that this A–P polarity may be required for determining left-right asymmetry.

**Figure 4:**
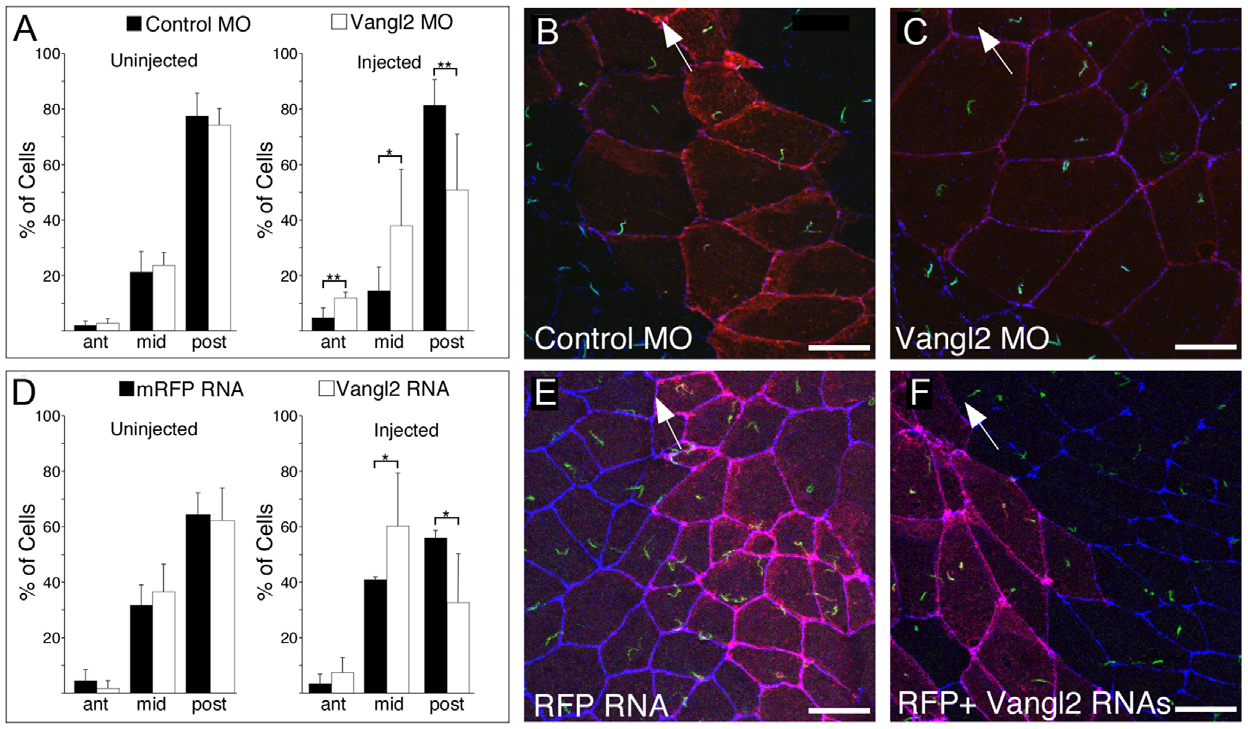
*Vangl2* Morpholino or RNA alters positioning of cilia in the GRP. (**A, D**) A clone of cells in the GRP was generated by injecting one dorsal blastomere of 8-cell stage embryos with *Vangl2* MO or a *Vangl2* RNA, along with *RFP* RNA as a tracer or as a control. At stages 17/18, the dorsal half of the embryo was removed, fixed, and stained with antibodies directed to ZO-1 and acetylated–tubulin to mark cell boundaries and cilia, respectively. GRP cilia both inside (injected) and outside (uninjected) the clone were scored based on the location in GRP cells that were divided into three equal zones (anterior, middle and posterior). Shown are data from a minimum of 3 GRPs where 100–200 injected and uninjected cells were scored. Asterisks (* = p<0.05, ** = p<0.01.) represent p-values obtained using a two-tailed t-test. (**B–C, E–F**) Shown are confocal images of the GRP injected with the *Vangl2* MO and *RFP* RNA (**C**), with Control MO and *RFP* RNA (**B**), with *Vangl2* and *RFP* RNAs (**F**) or with *RFP* RNA alone (**E**). RFP (red) marks the position of the injected clone, cilia (green) are stained with the acetylated-tubulin antibody, and cell boundaries (blue) are stained with an antibody to ZO-1. Anterior is indicated by direction of the arrow in **B, C, E, F**.

Because the low penetrance of obtaining phenotypically mutant *Vangl1gt/gt* homozygous embryos precluded analysis of mutant nodes, we chose to examine the role of PCP signaling in embryo lateralization in the homologous structure in *Xenopus*, the gastrocoel roof plate (GRP) [8]–[10]. In situ hybridization demonsrated that *Vangl2* is the predominantly expressed *Vang* gene in the GRP (**Fig. S4**), while little or no *Vangl1* expression could be detected (not shown). We have not been able to detect the localization of *Xenopus* VANGL2 protein in GRP cells, or in other tissues that are under control of a conserved PCP signaling mechanism based on strong functional evidence [29]. Nonetheless, to determine whether PCP might contribute to the correct posterior placement of motile cilia in the GRP, we chose to knock down VANGL2 function using morpholinos. Morpholinos were injected at the eight-cell stage, and the affected cells traced with co-injected RFP, thereby knocking down *Vangl2* expression within a subset of cells in both the deep and superficial mesoderm on the dorsal side. Injected embryos gastrulated normally, while the ciliated epithelium within the GRPs contained small cell clones with knocked down *Vangl2* adjacent to wild-type, uninjected cells (**Fig. 4**). In GRP cells, motile cilia normally relocate from a predominantly middle to a predominantly posterior position, and this posterior positioning is required to produce leftward flow [8]. In GRP cells with knocked down *Vangl2,* however, the distribution of cilia positioning is more central compared to the wild type distribution. This difference is statistically significant (**Fig. 4A**)**.** As overexpression of PCP proteins is known to dominantly perturb polarity [27], we also disrupted PCP by overexpressing full-length *Xenopus* VANGL2 in the GRP by mRNA injection, and, similar to knockdown of *Vangl2*, we again observed a more central positioning of motile cilia compared to wild type (**Fig. 4D–F**). Together, these results indicate that PCP in the frog GRP regulates the posterior positioning of motile cilia required for leftward flow.

**Figure 5:**
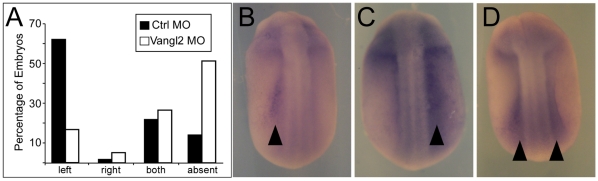
Vangl2 MO knockdown disrupts left-right patterning. (**A**) *Xenopus* embryos were injected with *Vangl2* MO, or Control MO alone, targeting one dorsal blastomere at the 8-cell stage to generate a clone of injected cells within the GRP. At stages 20/21, embryos were fixed and probed by in-situ hybridization with an antisense *Xenopus nodal-related1* (Xnr1) probe. Sidedness of staining was scored for at least 60 embryos for each condition. Data from one experiment are shown. (**B–D**) Shown are embryos injected with either *Vangl2* or Control MOs and then probed with *Xnr1* in-situ probe. *Xnr1* staining is evident on the left (**B**) right (**C**) or both (**D**) sides of the midline. Black arrowheads indicate staining.

To determine whether perturbing the posterior localization of motile cilia in the GRP affects left-right lateralization, we examined the expression of *Xenopus nodal* (*Xnr1–Xenopus nodal related 1*) mRNA in the *Vangl2* morpholino treated embryos. *Xnr1* is an early marker of lateralization that is normally expressed in the left lateral plate mesoderm (LPM) at late neurulation stage [8]. In our experiments, 58% of *Xenopus* embryos treated with control morpholinos had normal expression of *Xnr1*, in the left LPM, with smaller fractions showing expression bilaterally, on the right, or not at all (**Fig. 5**). In contrast, in the *Vangl2* morpholino treated embryos, lateralization was significantly impaired: only 9% showed *Xnr1* expression in the left LPM, whereas the fractions with right sided, bilateral and absent expression were increased relative to controls. In the morpholino-treated embryos, the largest class (∼53%) lacked expression of *Xnr1.* This result is similar to those previously reported upon knock-down of ciliary dynein heavy chain genes *dnah9* and *dnah5* or inhibition of the directional flow with methyl cellulose [8], [9]. It contrasts with results obtained in mice, in which disruption of genes important for L–R determination results in increased right and bilateral but not absent expression of markers such as *nodal* or *Pitx2* [2]. It is important to note that our *Vangl2* MO treated embryos did not display any signs of convergent extension defects, probably because *Vangl2* was knocked-down in only approximately one of eight cells, and these intermingled with wild-type cells within the deep layer of the mesoderm during intercalation. Our results demonstrate that the PCP gene *Vangl2* is required not only for posterior positioning of motile cilia in the GRP, but also for correct lateralization of *Xenopus* embryos.

## Discussion

Our results from mouse and frog, taken together, suggest a conserved function for PCP in establishing L-R asymmetry in vertebrates. In mouse, at least two polarity proteins, VANGL1 and PK2, become asymmetrically distributed along the A–P axis in the node, most likely prior to L–R asymmetry determination, by localizing to anterior cortical domains in central node cells. We suggest that the very early establishment of PCP in the ventral node and GRP triggers the proper posterior localization of motile cilia that is required for efficient leftward nodal flow [2], [10], [30]. Indeed, frog GRPs in which PCP is perturbed by knockdown or overexpression of *Vangl2* show substantially less posterior bias of cilia positioning, and these embryos fail to correctly lateralize. Results of other studies predict that this would impair directional fluid flow produced by nodal cilia [2], [30].

Consistent with these results, in another report, *Dishevelled (Dvl)* compound mutant mice lacking 5 of 6 copies of the three *Dvl* genes also show L–R laterality defects, and motile cilia in the node are mislocalized [31]. A DVL2-GFP showed that DVL2 protein, like VANGL1 and PK2, localizes to A–P cell boundaries in the ventral node, but to the posterior sides of cells [31], opposite to the apparent anterior localization of VANGL1 and PK2. DVL proteins are implicated in establishing the asymmetric cortical domains of PCP proteins [17], and have also been shown to be required for proper basal body and therefore cilia localization [32]. It is not clear from these results in which capacity DVL proteins function in the ventral node. Based on the conserved localization of PK, VANG and DVL proteins in nodal epithelium, with PK2 and VANGL1 localized opposite to DVL2, one would predict that PCP proteins operate through asymmetric cortical domains to establish molecular asymmetry by a mechanism similar to that described in flies [14], [15], which would, in turn, affect placement of cilia and thus regulate morphologic planar polarity. The functional importance of VANG in regulating PCP in the *Xenopus* GRP also highlights the likelihood that the evolutionarily conserved mouse and frog nodal structures share this mechanistic feature required for embryonic lateralization [10].

The signals that operate upstream of the core PCP proteins to provide anterior-posterior directionality to the planar polarization of the ventral node or GRP are unknown. Similarly, the mechanism by which the anterior-posteriorly organized core proteins control the posterior localization of motile cilia is unknown. However, our results indicate that anterior-posterior PCP is important for establishment of vertebrate L–R asymmetry. The conservation of this mechanism in the mouse ventral node and the *Xenopus* GRP is reminiscent of anterior/posterior localization of PCP proteins in other organisms, and reflects deep conservation of molecular and, most likely, functional anterior/posterior polarization in the evolution of Chordates, from Ascidian notochord to zebrafish gastrula dorsal mesoderm, notochord, and neural keel cells [33]–[35].

## Methods

### Generation of *Vangl1* Deficient Mice

The ES cell line, XL802, containing the beta-Geo insertion in the third intron of the *Vangl1* locus was purchased from BayGenomics (MMRRC at UC Davis, http://www.mmrrc.org/). The ES cells, genetically 129Ola, were injected into blastocysts isolated from C57BL/6 females (The Jaxon Laboratory) to generate chimeric males. Crosses between the chimeras and the C57BL/6 (Harlan, http://www.harlan.com/models/c57bl6.asp) females produced one F1 heterozygous male that was back-crossed with C57BL/6 females (Harlan) to generate a larger population of F2 heterozygous animals. Interbreeding of heterozygous animals produced subsequent generations of animals of mixed C57BL/6-129Ola background. Genotyping of 131 embryos extracted at E9.5 revealed that there were 35 (27%) *Vangl1gt/gt* homozygotes, 66 (50%) *Vangl1+/gt* heterozygotes, and 30 (23%) *Vangl1+/+* homozygous embryos. From 35 *Vangl1gt/gt* homozygotes, only 5 (14%) had a mutant turning phenotype. All animals were maintained and bred according to Institutional Animal Care and Use guidelines under protocols approved by the Administrative Panel on Laboratory Animal Care (APLAC) at Stanford University.

### Genotyping of *Vangl1* Deficient Mice

The precise beta-Geo insertion site was mapped by PCR to nucleotide position 239 of the third intron. Based on the insertion site, the following primers were used for PCR based genotyping of the obtained animals: VL1-Ex3F primer (5′-CCATCCAGGCTCCTGCTGGAG-3′) was used as a “forward” primer for genotyping of the wild type (WT) and the beta-Geo containing *Vangl1* locus, the VL1-Int3-2R primer (5′-CAGTCTCCAGATCTGTCCAGTCCCAAC-3′) was used as a “reverse” primer for the WT *Vangl1* locus, and the BGeoR3 primer (5′-CGTGTCCTACAACACACACTCCAACC-3′) was used as a “reverse” primer for the *Vangl1* knock-out locus. The PCR reactions were performed with Qiagen TaqMASTER Mix. The genotype of the obtained founder animals was further confirmed by Southern blotting (not shown).

### RT-PCR for *Vangl1-βGeo* and *Vangl1* WT mRNAs

Total RNA was isolated from adult cerebellum using Trisol reagent (Invitrogen). Vangl1-βGeo mRNA was detected using identical amounts of total RNA (300 ng) as template for each sample. The RT reaction was primed with BGeo-R1 primer (5′-GACAGTATCGGCCTCAGGAAGATCG-3′) and PCR reaction was carried out with Ex3F (5′-CCATCCAGGCTCCTGCTGGAG-3′) and BGeo-R1 primers. To detect Vangl1-WT mRNA, 300 ng of total RNA was used and the RT reaction was primed with an oligo-dT primer. 100 ng of each cDNA was used for the subsequent PCR reaction with 117-For (5′- CAGAGGGTCAGAAAAGTCTGTCACC-3′) and 996-Rev (5′-GCAATCATGGCTCGGGACTG-3′) primers. The specificity of the bands was confirmed with nested PCR using Ex3F and 943Rev (5′-CAGGCCAGCCATGTGCTTGG-3′) primers.

### Crosses between *Vangl1+/gt* and *Vangl2+/Lp* (*Lp/+*) Animals

The *Vangl2+/Lp* animals were maintained in the mixed 129P2Ola/C57BL6 background. The animals had highly curled tails and females had imperforate vaginas. To obtain reproductively capable females, the *Vangl2+/Lp* males were out-crossed into the CD1 background for a maximum of two generations. Crosses between the two mouse strains were carried out with the F1 and F2 generation of *Vangl1+/gt* animals.

### Cloning of the Mouse *Vangl* Genes

*Vangl1* and *Vangl2* genes were cloned by reverse-transcriptase-PCR from total RNA isolated from E18.5 CD-1 mouse embryos, using gene-specific primers designed according to the genomic sequence retrieved by the BLAT (BLAST-like Alignment Tool) program (University of California, Santa Cruz, http://genome.ucsc.edu/). The full-length cDNAs were cloned into eukaryotic expression vectors to generate either the YFP-tagged or untagged fusion constructs using the Invitrogen Gateway system (Carlsbad, CA).

### Production and Dharacterization of VANGL1 Antisera

To produce antisera to mouse VANGL1 protein, a cDNA fragment encoding the 207 C-terminal amino acids of VANGL1 was subcloned into the pATH10 vector to generate TrpE fusion protein for rabbit and rat immunization. This region was also cloned into the pET28a vector to produce histidine-tagged VANGL1 fragment, which was used for affinity purification of the antiserum. Western blotting and immunohistochemistry were used to determine specificity of the VANGL1 antibody (**Fig. S5**). Human epithelial cervical cancer (HeLa) cells, obtained from ATCC (cell line CCL2), were transfected with the mammalian expression plasmid pDS (negative control), or with the pDS plasmid encoding the full-length untagged or YFP-tagged VANGL1 or VANGL2 proteins, using FuGENE (Roche, Indianapolis, IN). Protein lysates were made 24 hours after transfection in a hypotonic buffer containing 10 mM Tris, pH 7.5, 50 mM KCl, 5 mM EDTA, 1% Triton X100, and Roche complete protease inhibitors and were analyzed by Western blotting. In addition, VANGL1 purified antibody was used on blots to detect endogenous VANGL1 protein produced by inner ear sensory epithelial patches. The lysates were prepared from dissected sensory epithelia using hypotonic buffer and were analyzed by Western blotting either before or after endoglycosidaseF treatment (Roche, Indianapolis, IN).

### Beta-Galactosidase Staining of Embryos

Mouse embryos were fixed in 4% paraformaldehyde in PBS, pH 7.4 for 20 minutes at 4°C, washed extensively in PBS, and stained with X-Gal (0.4 mg/ml X-gal, 10 mM potassium ferrocyanide, 10 mM potassium ferricyanide, 1 mM magnesium chloride in PBS) overnight at room temperature. After staining, embryos were fixed in 4% paraformaldehyde in PBS, pH 7.4 overnight at 4°C and were imaged as whole mounts (Leica MZFLIII dissecting scope).

### Immunostaining of Node-Stage Embryos

Node stage embryos were dissected in ice-cold PBS and fixed in the ice-cold 4% paraformaldehyde in PBS, pH 7.4 for 2 hours at 4 C. Embryos were washed 4 times, 30 minutes per wash, in PBS at 4C and then incubated in BLOCK (PBS +0.1% TX100 +2%BSA) for 1 hour at room temperature. Staining was carried with the primary antibodies, diluted in BLOCK, for 3–4 hours at room temperature or over night at 4C and was followed by 3 washes, 30 minutes each wash, in PBS/0.1% TX100 (PBT). Secondary antibodies, diluted in BLOCK, were added and incubated for 1–2 hours at room temperature. Embryos were washed 3–4 times, 30 minutes each wash, in PBT and then mounted for confocal imaging.

### *Xenopus laevis* Experiments

*Xenopus* embryos were obtained by in vitro fertilization using standard protocols. A synthetic *Vangl2* mRNA (1–5 ng) [29] was injected into one dorsal animal blastomere of eight-cell stage *Xenopus* embryos [36]. Cells derived from the injected blastomere were traced by co-injection of RNA encoding centrin fused to RFP (centrin-RFP) [37], or membrane-localized RFP [38]. At stage 17, the gastrocoel roof plate (GRP) was dissected away from the embryo [8] in Danilchik's buffer +0.1% BSA and then held under a piece of coverglass for fixation in 4% paraformaldehyde in Phosphate Buffered Saline (PBS) for 1 hour on ice followed by dehydration in 100% ethanol. GRPs were rehydrated, washed with PBS/0.1% TritonX-100 (PBT), and blocked with PBT containing 10% heat-inactivated normal goat serum (PBT/HIGS) for at least one hour. GRPs were incubated with primary antibody in blocking solution overnight as follows: Rabbit anti-ZO-1 (Zymed 1∶200), mouse monoclonal anti-acetylated tubulin (Sigma, 1∶1000). After washing, GRPs were incubated overnight in Cy2, or Cy5 labeled Goat anti-IgG of the appropriate species (all used at 1∶500, Jackson ImmunoResearch), washed in PBT and mounted in PVA/DABCO. Mounted embryos were imaged on a BioRad Radiance 2100 confocal mounted to a Zeiss inverted microscope using a 40× or 63× objective. Confocal images were false colored and z-stacks were compressed using NIH ImageJ. In some experiments, cilium location, based on centrin-RFP foci, was scored as being in the anterior, middle or posterior third of the cell in relation to the blastopore in each embryo. If centrin-RFP foci were not clearly evident, cilia were only scored if the entire cilium lay within a single one-third of the cell. In other experiments, cilium location was scored using acetylated tubulin, and cilia were only scored if the entire cilium lay within a single one-third of the cell. Data were gathered from 80–100 GRP cells marked with tracer as well as neighboring uninjected cells as a control, from at least 3 different embryos.

A morpholino (Genetools) directed against *Vangl2* initiation codon, as described previously [29], or a standard control morpholino [39], [40] was injected (20–30 ng; a concentration previously shown to produce a strong phenotype without toxicity) into one dorsal animal blastomere of eight-cell stage embryos in order to target the developing GRP [8]. Cilia localization was analyzed as above for *Vangl2* RNA injections. At stage 20–22, embryos were fixed and probed with an antisense probe directed against *Xenopus nodal-related1 (Xnr1)*, to mark the lateral plate mesoderm, using standard in-situ hybridization protocols [41]. Following labeling, experiments were blinded to the scorer and embryos were scored as having *Xnr1* expression on the left side, right side, both sides or staining absent.

### Antibodies

In this study, the following primary antibodies were used: rabbit anti-PK1, rabbit anti-PK2[21], rabbit and rat anti-VANGL1. These antibodies were generated in our labs. Commercially available antibodies used in this study were: mouse anti-spectrin (MAB1622, Chemicon, Temecula, CA), mouse anti-acetylated tubulin (T6793, Sigma, St. Louis, MO). Actin was stained with Alexa594-conjugated phalloidin (Invitrogen, CA). Secondary Antibodies coupled to Alexa 488, 594, or 633 dyes (Invitrogen) were used at 1∶500 dilution.

## Supporting Information

Figure S1 ***Vangl1*** **mutant mice. (A, B)** Map of the *Vangl1* locus in *Vangl1*-deficient mice, and genotyping by PCR. (**A**) Diagrams of the *Vangl1* genomic region containing a *β-Geo* insertion, the corresponding Vangl1-βGeo mRNA, and the resulting VANGL1-βGeo fusion protein. Remaining three exons and introns of the *Vangl1* gene are not drawn but are indicated with an arrow after exon 4. Positions of primers used for PCR based genotyping are marked (> and <). The *β-Geo* gene, together with the splice acceptor (SA) site and the polyA tail, is inserted at position 239 of the 3rd intron in the *Vangl1* locus of the XL802 ES cell line. The resulting VANGL1-βGeo fusion protein contains 70 N-terminal amino acids of the VANGL1 protein, and lacks all the trans-membrane domains (TM1-TM4) and the downstream C-terminal parts of the native VANGL1 protein. (**B**) PCR genotyping of a litter obtained by crossing two *Vangl1*+/− animals: the wild-type (WT) embryo genomic DNA produced only a 791 nucleotide fragment (embryo number 4, +/+), the *Vangl1* heterozygous embryos produced both the wild-type and the insert (411 nucleotides) bands (embryo number 2, +/−), and the *Vangl1gt/gt* homozygous embryos lack the wild-type and produced only the insert band (embryos number 1 and 3, −/−). (**C–F**) E17.5 *Vangl1+/gt*, *Vangl2+/Lp* double heterozygous animals have craniorachischisis. They are shorter than *Vangl1+/gt* heterozygous embryos or the wild-type littermates (**C**) and have closed eyelids (**C, D**). (**C–F**) Only double heterozygotes are stained with X-Gal, showing the distribution of VangL1 protein, and they have severe craniorachischisis.
(4.30 MB TIF)

Figure S2 **Cochleas isolated from** ***Vangl1+/gt*** **,** ***Vangl2+/Lp*** **trans-heterozygotes with severe craniorachischisis do not have significant PCP defects.** Cochleas isolated from the *Vangl1/Vangl2* double heterozygous embryos had 2.5 turns, just like the cochleas from the wild-type littermates (**A**). (**B–E**) Cell organization in the basal and apical regions of cochlea was similar in the mutants and in the wild-type animals. Therefore, the convergent extension phenotype that is characteristic of other PCP mutants including the *Vangl2−/−* animals was not detected in our *Vangl1/Vangl2* double heterozygotes with craniorachischisis. (**B, D**) Only minor polarity defects (misalignment of cells) were observed in the *Vangl1/Vangl2* double heterozygous mutants. 6 mutant cochleas were analyzed.
(2.86 MB TIF)

Figure S3 ***Vangl1gt/gt*** **adults that are viable and fertile produce both VL1-βGeo and VL1-WT mRNAs as well as the VL1-WT protein.** The low penetrance of the *Vangl1−/−* embryonic lethal phenotype prompted us to examine expression of the Vangl1-βGeo fusion and Vangl1 WT mRNAs in *Vangl1gt/gt* adult tissues. To do so, we prepared mRNA and protein samples from cerebellum and found that VL1 protein is abundantly expressed in this adult tissue (not shown). (**A**) Position of primers that were used for RT-PCR is marked (>, <). To detect the Vangl1-βGeo fusion mRNA, we used oligo-dT to obtain cDNA for subsequent PCR reaction with Ex3F and BGeo-R1 primers that produced a ∼480 nucleotide RT-PCR product. To detect Vangl1 WT mRNA, we used oligo-dT to obtain cDNA for subsequent PCR reaction with 117-For and 996-Rev primers. The ∼880 nucleotide PCR product would be obtained only if βGeo was spliced out and Vangl1-WT mRNA was produced. (**B**) Prior to RT-PCR, animals were re-genotyped and comparison was carried out between the wild-type (CD1) mouse, three *Vangl1+/gt* heterozygotes and three *Vangl1gt/gt* homozygotes. (**C**) RT-PCR for Vangl1-βGeo and Vangl1 WT mRNAs: CD1 animals made only Vangl1 WT mRNA. All other animals made varying amounts of Vangl1-βGeo and Vangl1 WT mRNAs. Similarly, they made different amounts of the VANGL1-WT protein which migrated as approximately 60 kD band when isolated from cerebellum (**D, D'**). (**E, E'**) Expression of LacZ was analyzed in *Vangl1+/gt* embryos. Example from two litters is shown (**E, E'**). (**E'**) A litter containing two *Vangl1gt/gt* embryos: the first embryo on the left is normal and the third embryo from the left has a turning phenotype. This example demonstrates that one *Vangl1gt/gt* animal was phenotypically “rescued” (first embryo from the left) and the other one was not, presumably by varying alternative splicing of the mutant transcript to produce varying levels of VANGL1 wild-type protein. Further, two *Vangl1+/gt* embryos from the same litter (**E'**), produced varying amounts of VANGL1-βGeo fusion protein. Staining of all embryos was performed at the same time and under identical conditions.
(2.97 MB TIF)

Figure S4 **Vangl2 RNA is expressed in the GRP. (A, B)** Shown are dissected GRPs from stage 16/17 embryos that were probed with digoxigenin labeled antisense probes against a-tubulin (A–A″) or Vangl2 RNA (b–b″). Dorsal views are shown in **A** and **B**, with ventral views showing expression in the GRP in **A'** and **B'**. After imaging, the dissected GRPs were cut in half and imaged from in cross-section. The ventral, or GRP side, is up in panels A″ and B″ and expression in the GRP is marked by arrows.
(1.46 MB TIF)

Figure S5 **Newly generated antibody to VANGL1 protein is highly specific.** Specificity of the VANGL1 antibody was tested by Western blotting (**A, B**). (**A**) Antibody to VANGL1 detects only the VANGL1 or the YFP-VANGL1 protein and not the VANGL2 or the YFP-VANGL2 protein in extracts of transfected HeLa cells. HeLa cells were transfected with vectors expressing VANGL1 (VL1), VANGL2 (VL2), as well as the corresponding YFP fusion proteins (YFP-VL1 and YFP-VL2). Lysates containing over-expressed proteins were analyzed by Western blots and compared to lysates of untransfected HeLa cells (–) or to vector-transfected cells (vector). Western blots were probed with the affinity-purified antibody to VL1 protein or with the antibody to GFP protein. Blots were also probed for tubulin as a loading control. (**B**) Antibody to VANGL1 detects a single band in extracts made from P3 vestibular sensory epithelia. The protein detected by the VANGL1 antibody has a higher molecular mass (∼100-kD) than the predicted ∼60-kD. Protein of the predicted size is detected after endoglycosidaseF treatment of lysates, demonstrating that glycosylation is the main reason for the observed discrepancy in size. The band observed in HeLa cell lysates transfected with VL1 is ∼60-kD.
(1.08 MB TIF)
