# Supplementary figures and images for: Planar Cell Polarity Enables Posterior Localization of Nodal Cilia and Left-Right Axis Determination during Mouse and Xenopus Embryogenesis
Source: PLoS One. 2010 Feb 2;5(2):e8999. doi: 10.1371/journal.pone.0008999 (PMC2814853; doi:10.1371/journal.pone.0008999)

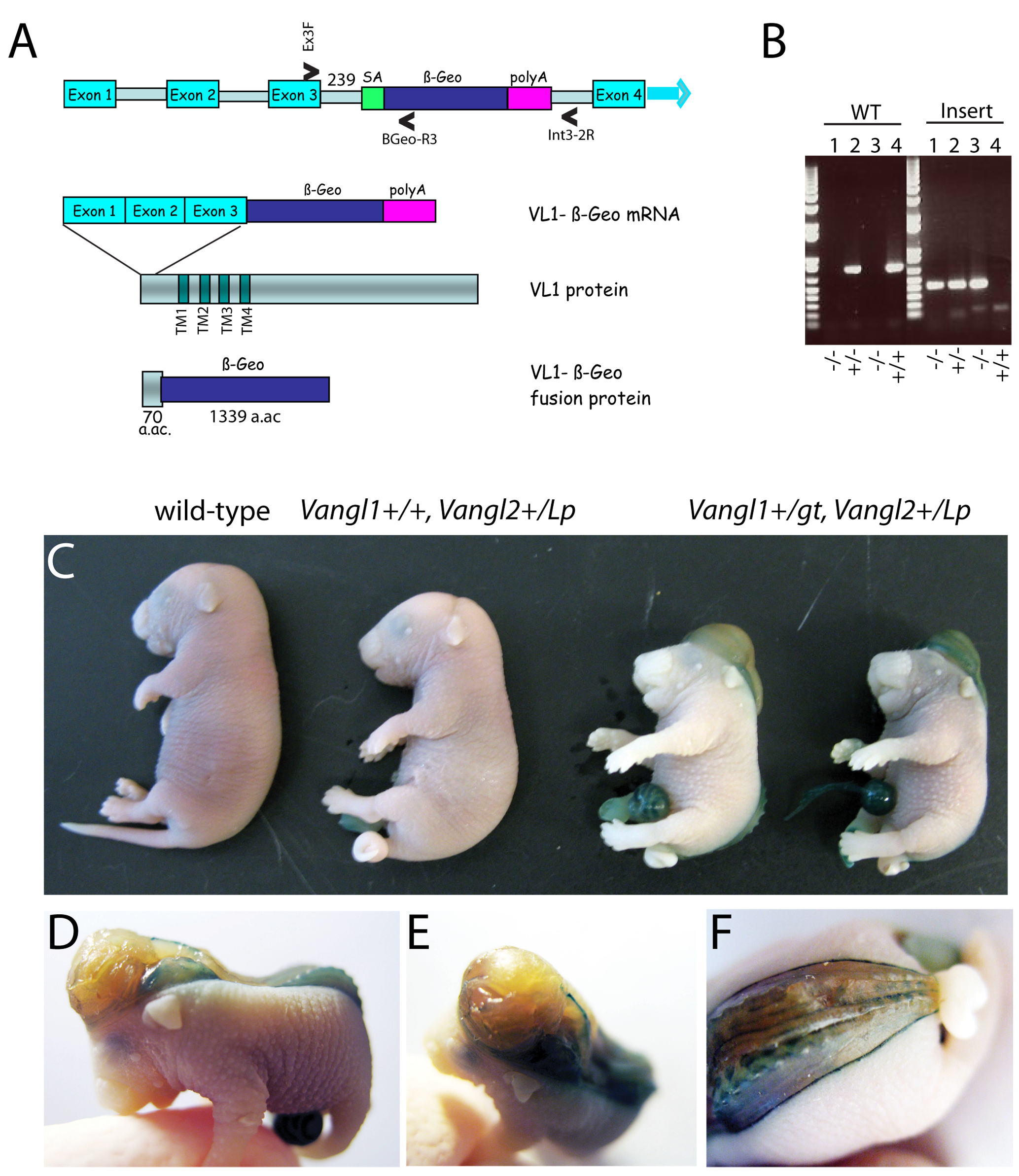

Supplement: Figure S1 — Vangl1 mutant mice. (A, B) Map of the Vangl1 locus in Vangl1-deficient mice, and genotyping by PCR. (A) Diagrams of the Vangl1 genomic region containing a β-Geo insertion, the corresponding Vangl1-βGeo mRNA, and the resulting VANGL1-βGeo fusion protein. Remaining three exons and introns of the Vangl1 gene are not drawn but are indicated with an arrow after exon 4. Positions of primers used for PCR based genotyping are marked (> and <). The β-Geo gene, together with the splice acceptor (SA) site and the polyA tail, is inserted at position 239 of the 3rd intron in the Vangl1 locus of the XL802 ES cell line. The resulting VANGL1-βGeo fusion protein contains 70 N-terminal amino acids of the VANGL1 protein, and lacks all the trans-membrane domains (TM1-TM4) and the downstream C-terminal parts of the native VANGL1 protein. (B) PCR genotyping of a litter obtained by crossing two Vangl1+/− animals: the wild-type (WT) embryo genomic DNA produced only a 791 nucleotide fragment (embryo number 4, +/+), the Vangl1 heterozygous embryos produced both the wild-type and the insert (411 nucleotides) bands (embryo number 2, +/−), and the Vangl1gt/gt homozygous embryos lack the wild-type and produced only the insert band (embryos number 1 and 3, −/−). (C–F) E17.5 Vangl1+/gt, Vangl2+/Lp double heterozygous animals have craniorachischisis. They are shorter than Vangl1+/gt heterozygous embryos or the wild-type littermates (C) and have closed eyelids (C, D). (C–F) Only double heterozygotes are stained with X-Gal, showing the distribution of VangL1 protein, and they have severe craniorachischisis. (4.30 MB TIF) [file pone.0008999.s001.tif]

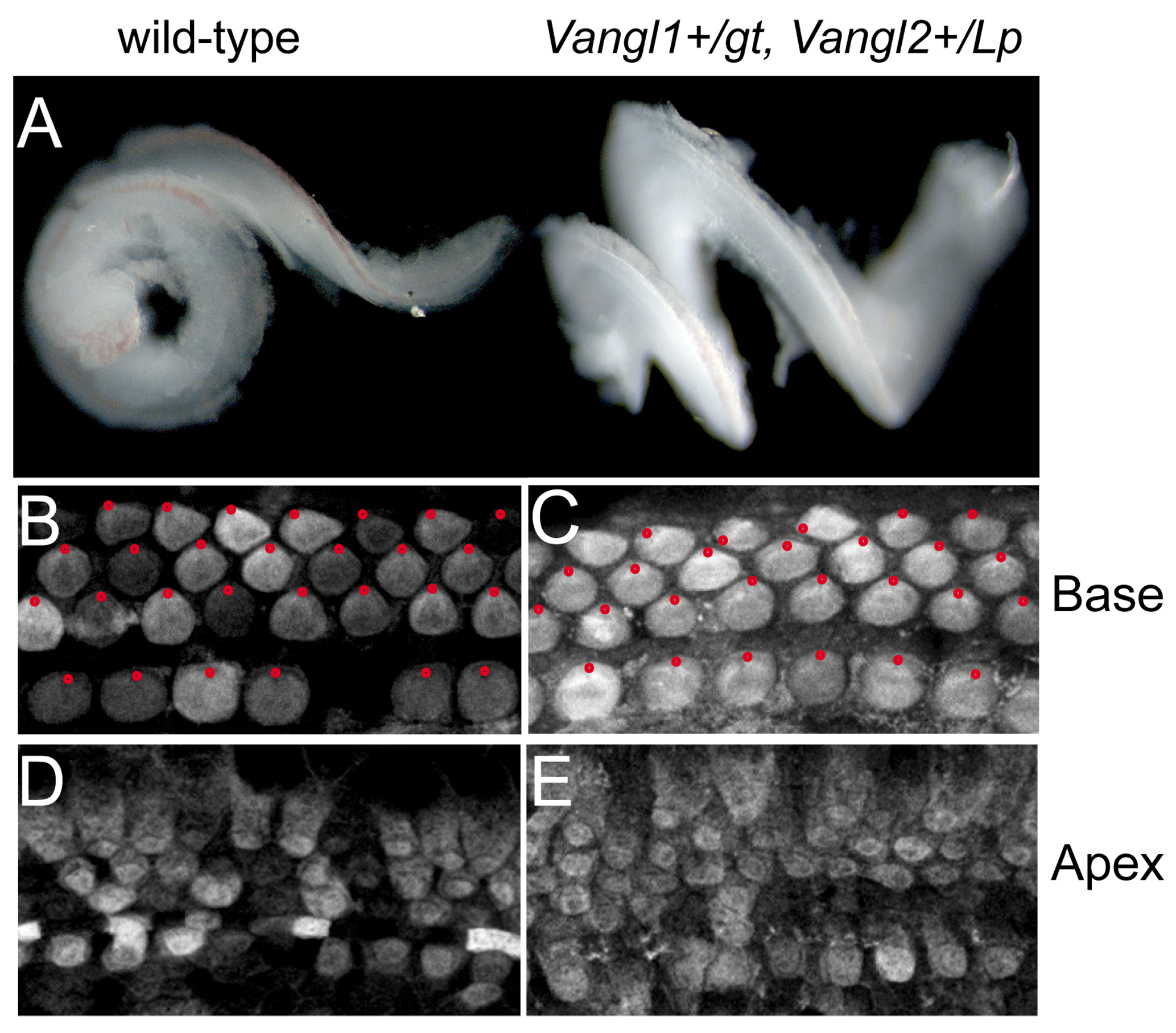

Supplement: Figure S2 — Cochleas isolated from Vangl1+/gt , Vangl2+/Lp trans-heterozygotes with severe craniorachischisis do not have significant PCP defects. Cochleas isolated from the Vangl1/Vangl2 double heterozygous embryos had 2.5 turns, just like the cochleas from the wild-type littermates (A). (B–E) Cell organization in the basal and apical regions of cochlea was similar in the mutants and in the wild-type animals. Therefore, the convergent extension phenotype that is characteristic of other PCP mutants including the Vangl2−/− animals was not detected in our Vangl1/Vangl2 double heterozygotes with craniorachischisis. (B, D) Only minor polarity defects (misalignment of cells) were observed in the Vangl1/Vangl2 double heterozygous mutants. 6 mutant cochleas were analyzed. (2.86 MB TIF) [file pone.0008999.s002.tif]

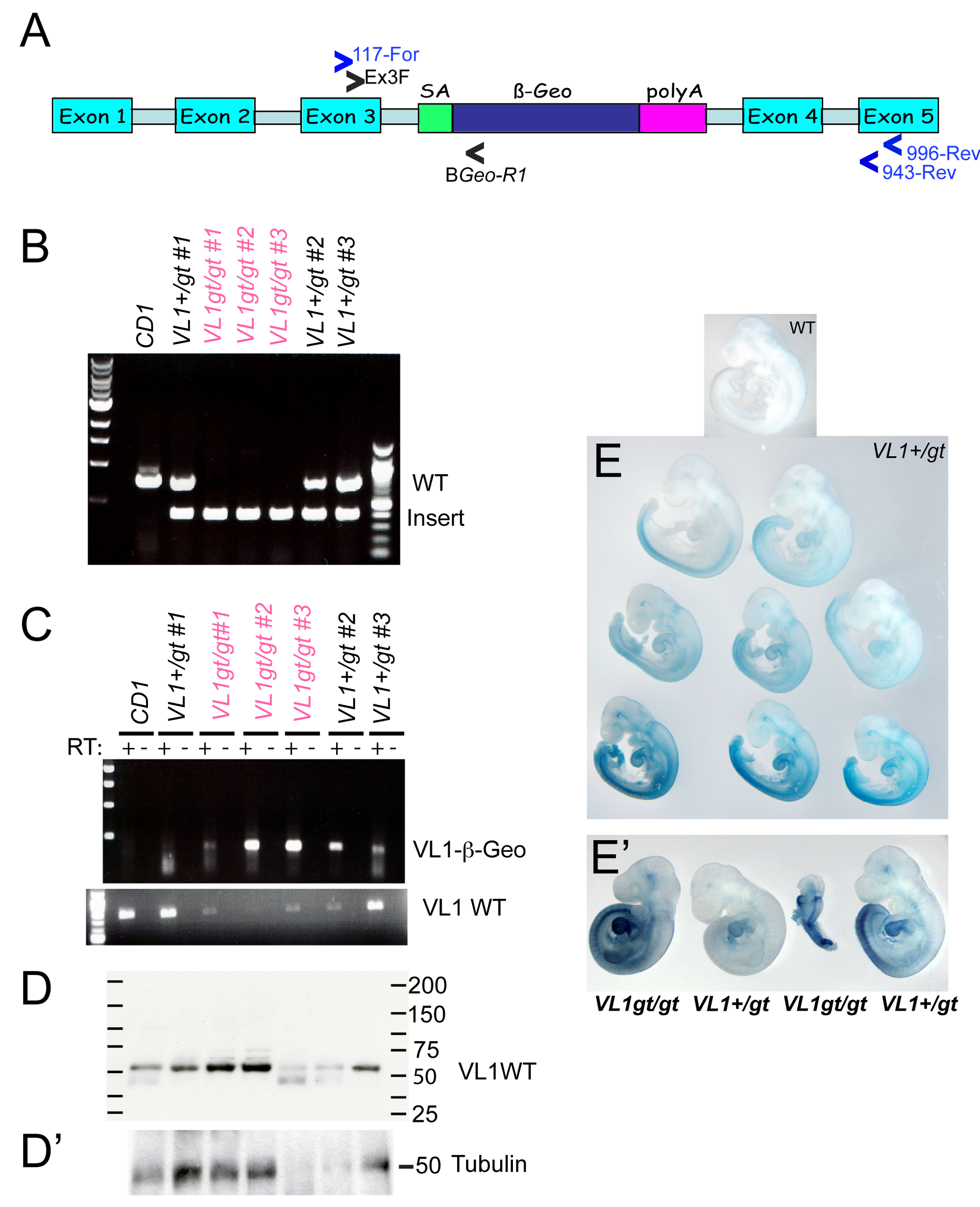

Supplement: Figure S3 — Vangl1gt/gt adults that are viable and fertile produce both VL1-βGeo and VL1-WT mRNAs as well as the VL1-WT protein. The low penetrance of the Vangl1−/− embryonic lethal phenotype prompted us to examine expression of the Vangl1-βGeo fusion and Vangl1 WT mRNAs in Vangl1gt/gt adult tissues. To do so, we prepared mRNA and protein samples from cerebellum and found that VL1 protein is abundantly expressed in this adult tissue (not shown). (A) Position of primers that were used for RT-PCR is marked (>, <). To detect the Vangl1-βGeo fusion mRNA, we used oligo-dT to obtain cDNA for subsequent PCR reaction with Ex3F and BGeo-R1 primers that produced a ∼480 nucleotide RT-PCR product. To detect Vangl1 WT mRNA, we used oligo-dT to obtain cDNA for subsequent PCR reaction with 117-For and 996-Rev primers. The ∼880 nucleotide PCR product would be obtained only if βGeo was spliced out and Vangl1-WT mRNA was produced. (B) Prior to RT-PCR, animals were re-genotyped and comparison was carried out between the wild-type (CD1) mouse, three Vangl1+/gt heterozygotes and three Vangl1gt/gt homozygotes. (C) RT-PCR for Vangl1-βGeo and Vangl1 WT mRNAs: CD1 animals made only Vangl1 WT mRNA. All other animals made varying amounts of Vangl1-βGeo and Vangl1 WT mRNAs. Similarly, they made different amounts of the VANGL1-WT protein which migrated as approximately 60 kD band when isolated from cerebellum (D, D'). (E, E') Expression of LacZ was analyzed in Vangl1+/gt embryos. Example from two litters is shown (E, E'). (E') A litter containing two Vangl1gt/gt embryos: the first embryo on the left is normal and the third embryo from the left has a turning phenotype. This example demonstrates that one Vangl1gt/gt animal was phenotypically “rescued” (first embryo from the left) and the other one was not, presumably by varying alternative splicing of the mutant transcript to produce varying levels of VANGL1 wild-type protein. Further, two Vangl1+/gt embryos from the same litter (E'), produced varying amount [file pone.0008999.s003.tif]

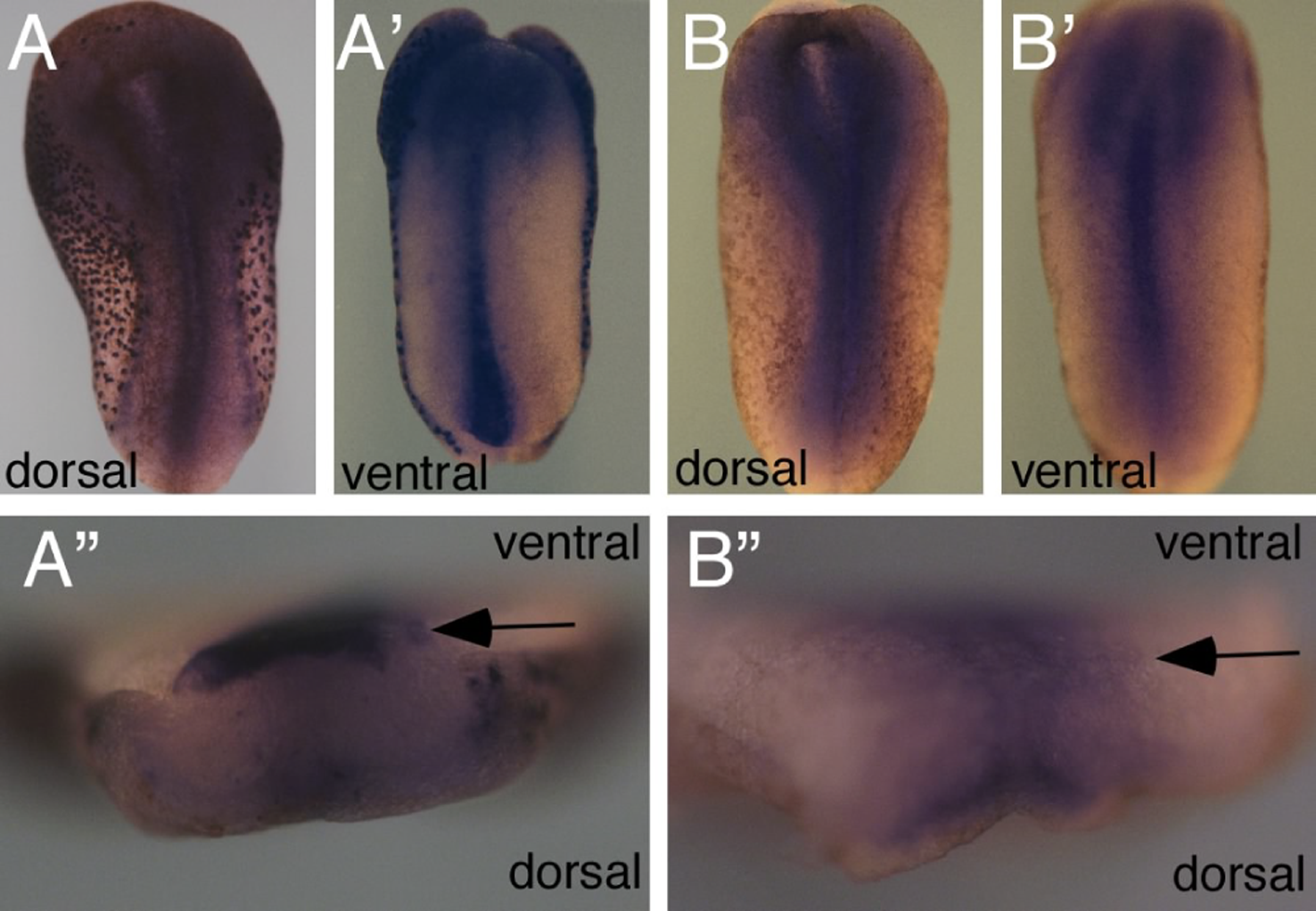

Supplement: Figure S4 — Vangl2 RNA is expressed in the GRP. (A, B) Shown are dissected GRPs from stage 16/17 embryos that were probed with digoxigenin labeled antisense probes against a-tubulin (A–A″) or Vangl2 RNA (b–b″). Dorsal views are shown in A and B, with ventral views showing expression in the GRP in A' and B'. After imaging, the dissected GRPs were cut in half and imaged from in cross-section. The ventral, or GRP side, is up in panels A″ and B″ and expression in the GRP is marked by arrows. (1.46 MB TIF) [file pone.0008999.s004.tif]

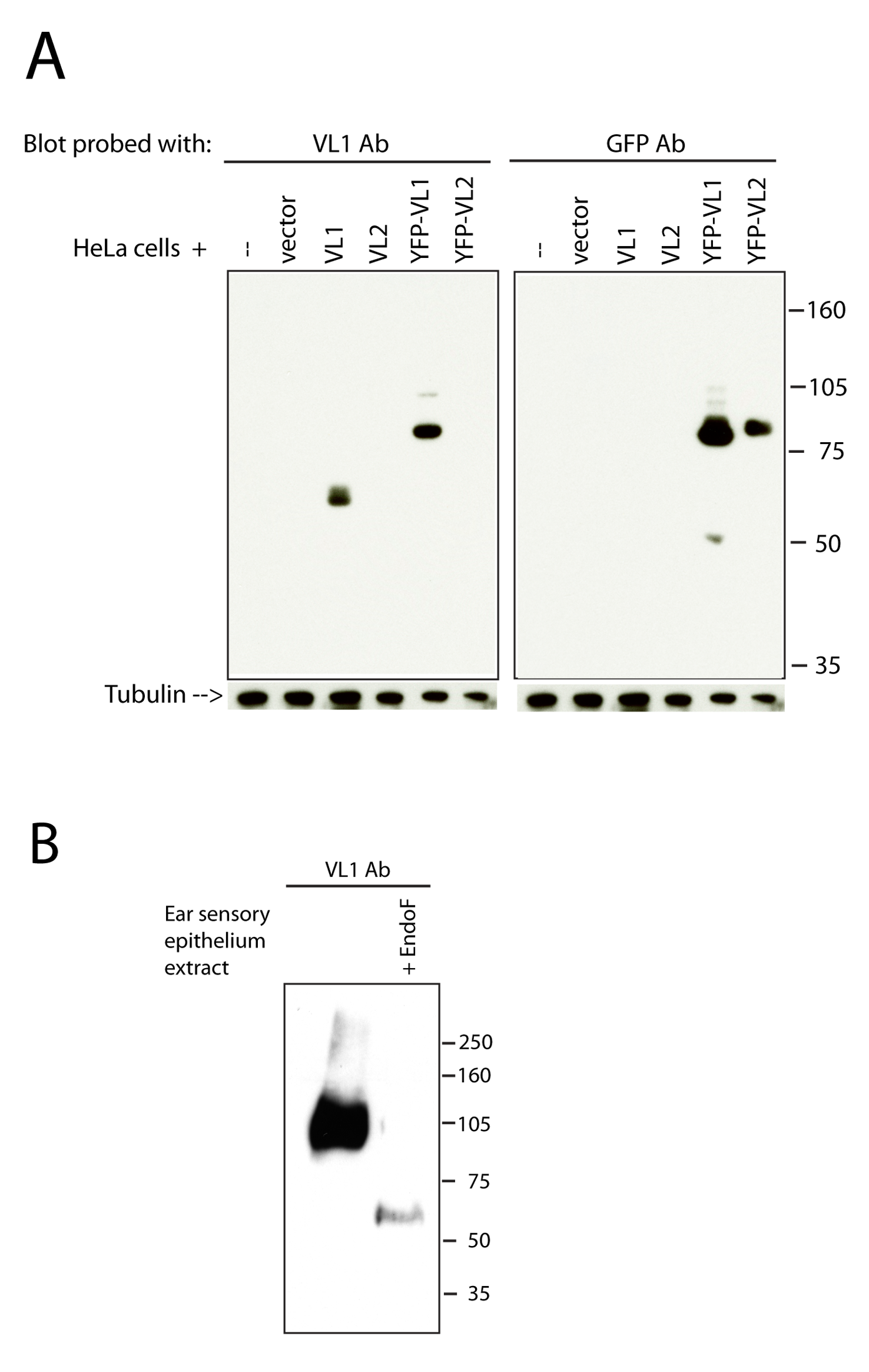

Supplement: Figure S5 — Newly generated antibody to VANGL1 protein is highly specific. Specificity of the VANGL1 antibody was tested by Western blotting (A, B). (A) Antibody to VANGL1 detects only the VANGL1 or the YFP-VANGL1 protein and not the VANGL2 or the YFP-VANGL2 protein in extracts of transfected HeLa cells. HeLa cells were transfected with vectors expressing VANGL1 (VL1), VANGL2 (VL2), as well as the corresponding YFP fusion proteins (YFP-VL1 and YFP-VL2). Lysates containing over-expressed proteins were analyzed by Western blots and compared to lysates of untransfected HeLa cells (–) or to vector-transfected cells (vector). Western blots were probed with the affinity-purified antibody to VL1 protein or with the antibody to GFP protein. Blots were also probed for tubulin as a loading control. (B) Antibody to VANGL1 detects a single band in extracts made from P3 vestibular sensory epithelia. The protein detected by the VANGL1 antibody has a higher molecular mass (∼100-kD) than the predicted ∼60-kD. Protein of the predicted size is detected after endoglycosidaseF treatment of lysates, demonstrating that glycosylation is the main reason for the observed discrepancy in size. The band observed in HeLa cell lysates transfected with VL1 is ∼60-kD. (1.08 MB TIF) [file pone.0008999.s005.tif]
